# Cortactin promotes colorectal cancer cell proliferation by activating the EGFR-MAPK pathway

**DOI:** 10.18632/oncotarget.13652

**Published:** 2016-11-26

**Authors:** Xiaojian Zhang, Kun Liu, Tao Zhang, Zhenlei Wang, Xuan Qin, Xiaoqian Jing, Haoxuan Wu, Xiaopin Ji, Yonggang He, Ren Zhao

**Affiliations:** ^1^ Department of Surgery, Ruijin Hospital, Shanghai Jiao Tong University School of Medicine, Shanghai, People's Republic of China; ^2^ Shanghai Institute of Digestive Surgery, Shanghai, People's Republic of China; ^3^ Department of Surgery, Ruijin Hospital North, Shanghai Jiao Tong University School of Medicine, Shanghai, People's Republic of China; ^4^ Department of Surgery, Henan Cancer Hospital, The Affiliated Cancer Hospital of Zhengzhou University, Zhengzhou, Henan Province, People's Republic of China

**Keywords:** colorectal cancer, cortactin(CTTN), epidermal growth factor receptor (EGFR), MAPK, c-Cbl

## Abstract

Cortactin (CTTN) is overexpressed in various tumors, including head and neck squamous cell carcinoma and colorectal cancer (CRC), and can serve as a biomarker of cancer metastasis. We observed that CTTN promotes cancer cell proliferation *in vitro* and increases CRC tumor xenograft growth *in vivo*. CTTN expression increases EGFR protein levels and enhances the activation of the MAPK signaling pathway. CTTN expression also inhibits the ubiquitin-mediated degradation of EGFR by suppressing the coupling of c-Cbl with EGFR. CoIP experiments indicate CTTN can interact with c-Cbl in CRC cells. These results demonstrate that CTTN promotes the proliferation of CRC cells and suppresses the degradation of EGFR.

## INTRODUCTION

Colorectal cancer (CRC) has become the third most common cancer in men (746,000 cases, 10.0% of the total), and the second most common in women (614,000 cases, 9.2% of the total) worldwide. Annually, colorectal cancer has led to about 694,000 deaths (8.5% of the total) [1]. Colorectal tumorigenesis follows the “multi-step adenoma–carcinoma sequence.” This sequence includes a series of genetic mutations and epigenetic alterations leading to genetic instability, like APC, RAS, p53, and the EGFR signaling pathway [2–4]. Defining these molecular alterations will help guide treatment and improve clinical care.

Cortactin (CTTN) is a substrate of v-SRC. When it is bound with actin, this activates the Arp2/3 complex, modulation of cytoskeleton formation, and debranching of dendritic actin under the plasma [5, 6]. CTTN is overexpressed in many tumors, across several cancers: head and neck, gastric, hepatocellular, and colorectal [7]. While Cortactin stimulates metastasis of cancer cells [8, 9], CTNN alsos inhibit down-regulation of the EGFR and MET signaling pathways [10–12]. Epidermal growth factor receptor (EGFR) is often overexpressed or mutated in a variety of cancers, like lung cancer and colorectal cancer [13, 14], and this leads to enhanced activation and tumorigenesis. The aim of this study is to investigate the biological role of CTTN in CRC and related molecules in the EGFR pathway.

## RESULTS

### CTTN is highly expressed in CRC tissues and correlates with pathological stage

**Figure 1:**
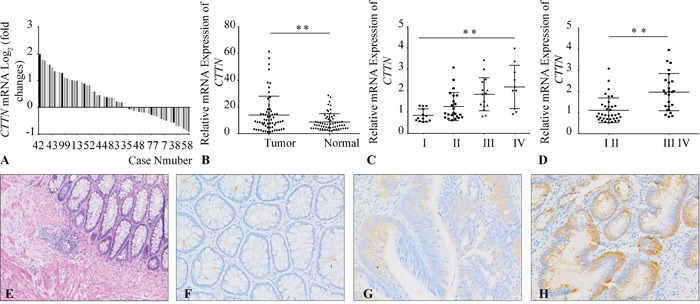
Overexpression of CTTN in CRC tissues **A.** The mRNA levels of *CTTN* in 61 paired CRC and matched non-tumor tissues were determined by qPCR. The data is expressed as the log_2_ fold change (ΔCt [Tumor/Non.]). **B.** Overexpression of *CTTN* in CRC compared with non-tumor tissues according to qPCR results. **C, D.** The *CTTN* mRNA overexpression in CRC tissues correlates with the tumor stage. **E.** The HE staining of normal colorectal tissue. **F.** Immunohistochemical staining of CTTN in normal colorectal tissue. **G, H.** Representative images of weak or strong intensity staining of CTTN in CRC. (*P < 0.05, images were taken under 200×magnification).

**Figure 2:**
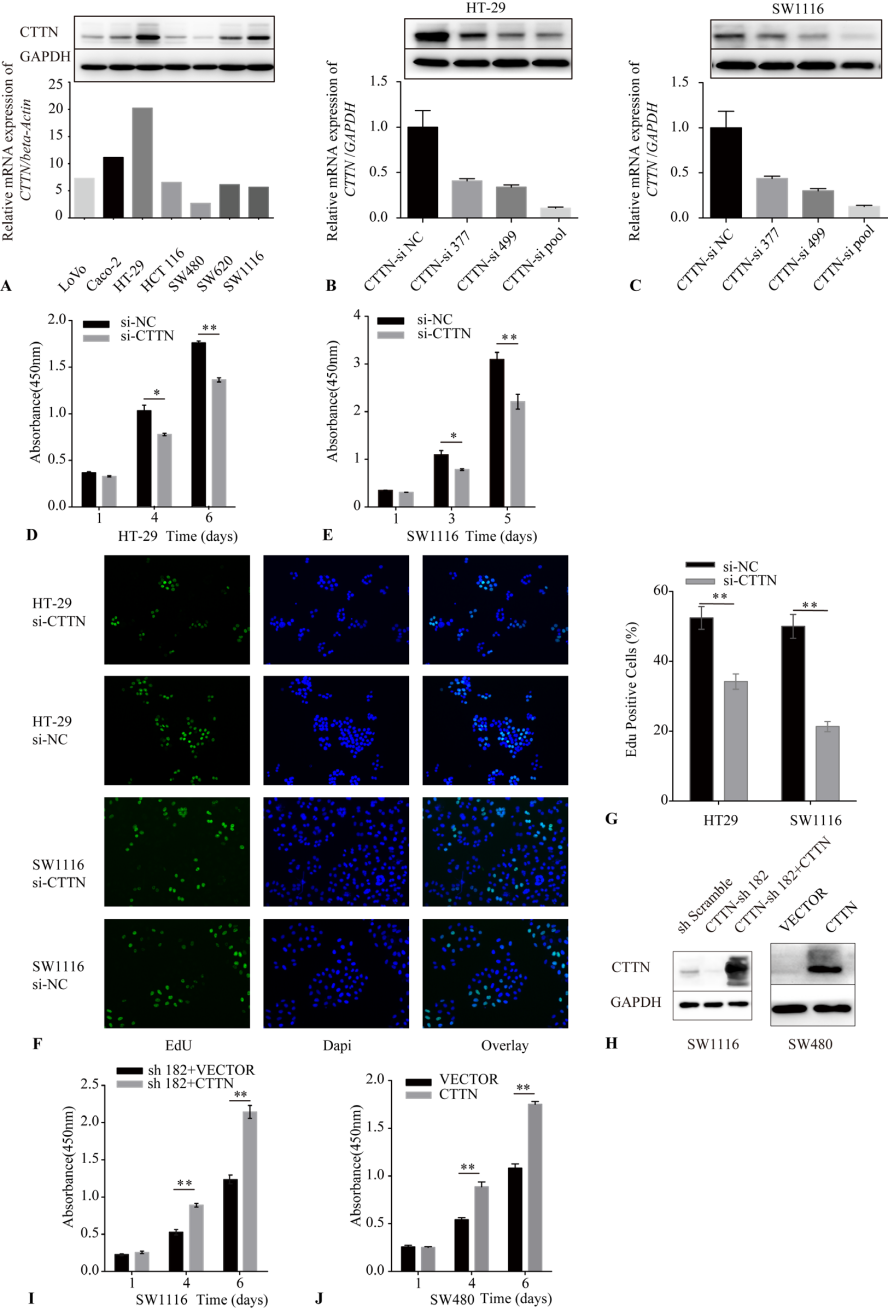
Downregulation of CTTN reduces cancer cell proliferation **A.** CTTN expression in seven colorectal cancer cell lines was examined by qRT-PCR and western blot (WB). **B, C.** The validation of CTTN-siRNA mediated knockdown of CTTN in HT-29 and SW1116 cells by qRT-PCR and WB. **D, E.** A representative result of the Cell Counting Kit-8 (CCK-8) assay for the effects of CTTN-si pool on the growth of HT-29 and SW1116 cells *in vitro*. **F.** Representative results of the EdU incorporation assay for the effects of CTTN-si pool on the proliferation of HT-29 and SW1116 cell *in vitro*. **G.** The proportion of EdU-staining positive cells in the CTTN-si pool group and NC group. **H.** The detection of rescue expression of CTTN in SW1116 CTTN-sh182 cells and overexpression of CTTN in SW480 cells. **I, J.** Representative results of the CCK-8 assay for the effects of rescue expression of CTTN in SW1116 CTTN-sh182 cells and overexpression of CTTN in SW480 cells. (*P < 0.05, images were taken under 40×magnification).

To explore the association of CTTN expression with clinicopathological parameters in CRC, we examined the mRNA expression of *CTTN* in 61 pairs CRC tissues by qRT-PCR. There is about 52% of CRC specimens with *CTTN* mRNA upregulation (35/61) (Figure 1A), and the relative CTTN mRNA expression in CRC is higher than matched normal tissue (Figure 1B). There is a positive correlation between CTTN mRNA expression and CRC tumor stage (Figure 2C, 2D), and *CTTN* expression is not related to gender, age, or tumor site (Supplementary information, Table S4). We further examined CTTN protein expression in CRC and normal mucosa by immunohistochemistry. The weak staining of CTTN in normal mucosa is present in cytoplasm (Figure 1F, 1G, 1H, Supplementary Figure S4), while the expression of CTTN in CRC is much higher than the normal tissue with a strong staining. The above results imply that increased CTTN expression may be associated with the tumor progression of CRC, thus providing clues to further analyze its biological function and molecular mechanism in CRC progression.

### Suppression of CTTN inhibits cancer cell proliferation *in vitro*

We made a CTTN expression assay in seven colorectal cancer cell lines using qRT-PCR and western blot (Figure 2A). CTTN expression was highest in the SW1116 and HT-29 cell lines, while the expression of mRNA and protein was much less in the SW480 line. Therefore, we adopted the SW1116, HT-29, and SW480 cell lines as the cellular models to explore the function of CTTN *in vitro*.

We used RNA interference (RNAi) to suppress CTTN expression. A pool of two different siRNAs including CTTN-siRNA 377 and siRNA 499 were applied, and the efficiency of each siRNA, or the pool of siRNA, was validated by qRT-PCR and western blot (Figure 2B, 2C). The effect of knockdown of CTTN on the growth of HT-29 and SW1116 was measured by the Cell Counting Kit-8 (CCK-8) assay. The reduction of CTTN inhibited the growth of these two cell lines (Figure 2D, 2E).

To ascertain whether CTTN could promote cancer cell proliferation, we labeled cancer cells with the thymidine analog EdU after siRNA pool transfection to monitor the activity of DNA synthesis (Figure 2F). The percentage of cells labeled with EdU in the CTTN-si group is lower than the control group (Figure 2G). To further verify the effect of CTTN on CRC cells, we upregulated CTTN expression by lentiviral infection of CTTN-ORF plasmid in SW480 and SW1116-sh182 (in which the shRNA targets the 3′UTR of CTTN mRNA) cell lines. CTTN overexpression was confirmed by immunoblotting (Figure 2H). We measured the growth rate of SW480-CTTN and SW1116-sh182+CTTN cell lines by CCK-8. These data demonstrate that CTTN overexpression promotes the growth of CRC cells (Figure 2I, 2J).

### CTTN enhances CRC cell clonogenicity *in vitro*

**Figure 3:**
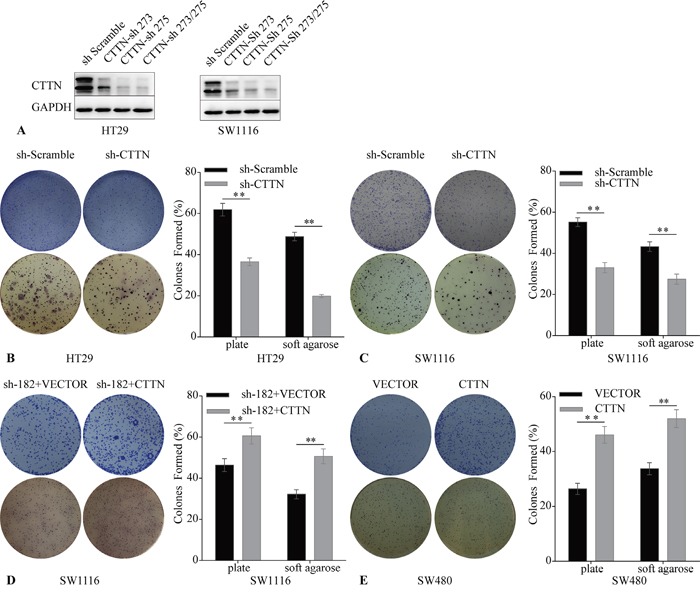
Downregulation of CTTN induces reduction of CRC cell clonogenicity **A.** The stable downregulation of CTTN by lentiviral infection was evaluated by WB. **B, C.** The knockdown of CTTN suppresses HT29 and SW1116 colony formation. **D, E.** CTTN overexpression increases the colony formation of SW480 and SW1116 CTTN-sh 182 cells. (*P < 0.05).

Transformed tumor cells can self-renew in an anchorage-independent or anchorage-dependent manner. This clonogenic ability is positively correlated with the metastasis of cancer cells [15]. To assay the effect of CTTN on clonogenic ability of CRC cells, we downregulated CTTN expression in HT-29 and SW1116 cells via lentiviral infection. We designed a plasmid including shRNA-273 and shRNA-275, targeting two different sites of *CTTN'* CDS (Figure 3A). The results of plate clone formation and soft agar clonogenic assays showed that more, and larger, colonies formed in the control group than the corresponding CTTN-sh group (Figure 3B, 3C). The SW480 CTTN and SW1116-sh182+CTTN cell lines, which overexpress CTTN, formed more colonies than the control group (Figure 3D, 3E). These results suggest CTTN enhances the clonogenic ability of CRC cell lines, and this data is consistent with previous HNSCC studies [11].

### Downregulation of CTTN suppresses tumor growth *in vivo*

**Figure 4:**
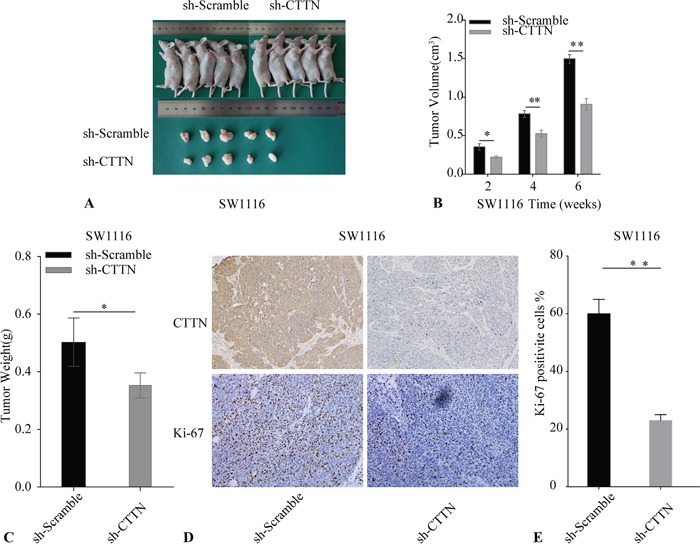
Knockdown of CTTN inhibits CRC cell growth *in vivo* **A.** The reduction of CTTN expression in SW1116 cells suppresses tumor growth. **B.** Tumor volume was examined every 2 weeks after implantation of SW1116 sh-CTTN and sh-scramble cells. **C.** The weight of tumor tissue in SW1116 CTTN-sh and control groups. **D, E.** Tumor sections were stained with Ki-67 and CTTN, with an original magnification of ×100 (*P < 0.05).

To further ascertain the proliferation role of CTTN *in vivo*, we adopted a xenograft animal model using the BALB/c nude mouse. SW1116 sh-Scramble and SW1116 sh-CTTN transfected cells were subcutaneously injected into mice. Knockdown of CTTN in SW1116 suppresses tumor growth *in vivo* (Figure 4A). Additionally, tumor growth rate, size, and weight in the sh-CTTN group is much lower than the control group (Figure 4B, 4C). Immunohistochemical staining of the tumor with Ki-67 confirms the inhibition of cancer cell proliferation in sh-CTTN group (Figure 4D). Tumor tissues of the sh-CTTN group have a lower percentage of Ki-67 staining (Figure 4E). The downregulation of CTTN is responsible for the suppression of tumor growth *in vivo*.

### CTTN enhances the activation of MAPK pathway

CTTN promotes cancer cell mobility [16–19], and Timpson's research suggested CTTN inhibited EGFR ubiquitination and degradation in HNSCC [10, 11]. We found the EGFR protein level in the CTTN-sh group was lower than the corresponding control group when the CRC cells were conventionally cultured. CTTN overexpression also increased the level of EGFR (Supplementary Figure S1). Analysis of EGFR protein levels and ERK activation over a prolonged time course were quantified by densitometry (Supplementary Figure S5).

Downstream of the EGFR signaling pathway includes the Ras/Raf/mitogen-activated protein kinase pathway, Phosphatidylinositol 3-kinase/Akt pathway, Src kinase pathway, and Signal transducers and activators of the transcription pathway [21, 22]. We used a CST EGFR PathScan array to explore the possible molecular mechanisms involved in the proliferative effect of CTTN in CRC cells. We stimulated the cell lysate from the SW1116 sh-CTTN and control cells with 10 ng/mL of EGF for 30 minutes, and found that the phosphorylation levels of ERK and PLCγ1 Ser1248 decreased in the CTTN-sh group (Supplementary Figure S2).

**Figure 5:**
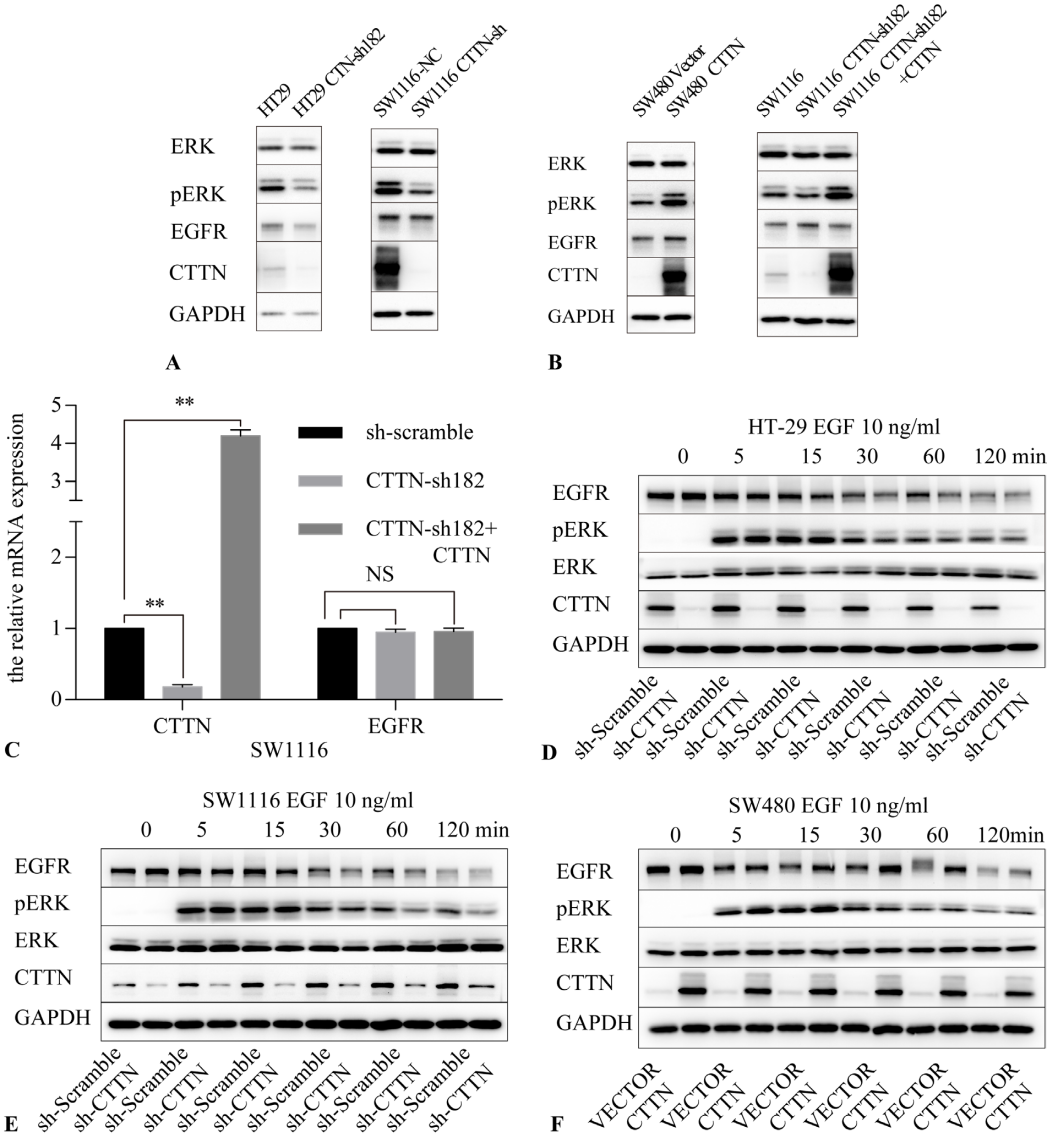
CTTN expression activates the MAPK pathway **A.** The knockdown of CTTN in HT-29 and SW1116 cells attenuates the phosphorylation level of ERK. **B.** The rescue expression of CTTN in SW1116 or overexpression of CTTN in SW480 cells increases the amount of EGFR and the phosphorylation level of ERK. **C.** mRNA analysis of EGFR after CTTN knockdown or rescue expression in SW1116 cells (n=3; Student t test, *P < 0.05, **P < 0.01). **D, E.** Ligand-induced down-regulation of EGFR was increased in CTTN-depleted cells. Serum-starved control or CTTN-sh CRC cells were pretreated with10 μM of cycloheximide for 1 h, followed by stimulation with 10 ng/mL EGF for the indicated time. **F.** Ligand-induced down-regulation of EGFR was compromised in SW480 cells with overexpressed CTTN. Similar results were obtained in three different experiments.

We further confirmed that knockdown of CTTN in HT29 and SW1116 inhibited ERK phosphorylation (Figure 5A), and the rescue expression of CTTN restored ERK phosphorylation after cells were stimulated with 10 ng/mL EGF for 30 minutes. Similarly, CTTN overexpression in SW480 increased ERK phosphorylation (Figure 5B). We also inhibited the ERK1/2 pathway by U0126, a specific ERK1/2 inhibitor. The CTTN-overexpressing cells of SW1116 and SW480 were cultured in medium containing U0126 for the indicated time. Tumor cells treated with U0126 had a lower level of p-ERK compared with the control group (Supplementary Figure S3A), and they proliferated at a slower rate and formed less colonies (Supplementary Figure S3B, S3C). This suggests that the MAPK signaling pathway is partially responsible for CTTN-induced cell proliferation. These data suggest CTTN promotes CRC cell proliferation by increasing ERK phosphorylation and activation of the MAPK pathway.

To explore the mechanisms of EGFR protein level changes under *CTTN* depletion, we first used qPCR analysis to assess EGFR mRNA synthesis following CTTN knockdown. We found that inhibition or rescue expression of CTTN did not interfere with EGFR mRNA levels (Figure 5C). This finding suggests that CTTN may regulate EGFR expression at a posttranslational level.

Next we determined the effect of CTTN on EGF-induced EGFR down-regulation in CRC cells. Cells were stimulated with 10 ng/mL EGF after starvation for 16 hours. To block protein synthesis, the cells were pretreated with cycloheximide for one hour. The cells were harvested at different time points for further immunoblotting analyses. The experimental results indicate the knockdown of CTTN reduces the protein level of EGFR. Down-regulation of EGFR was rapid, with only about 10% of the receptor remaining after 60 minutes in the CTTN-sh group of HT-29 and SW1116 compared with base level (Figure 5D, 5E). EGFR down-regulation was slower in SW480 cells, with about 60% of the EGFR remaining after 60 minutes (Figure 5F). CTTN expression maintained EGF-induced ERK activation. Because we blocked the protein synthesis in the beginning of the experiments, these results suggest that CTTN may increase EGFR degradation induced by EGF. In CRC cells, CTTN expression attenuates EGF-induced down-regulation of EGFR.

### EGF potentiates proliferative effect of CTTN in CRC cells

**Figure 6:**
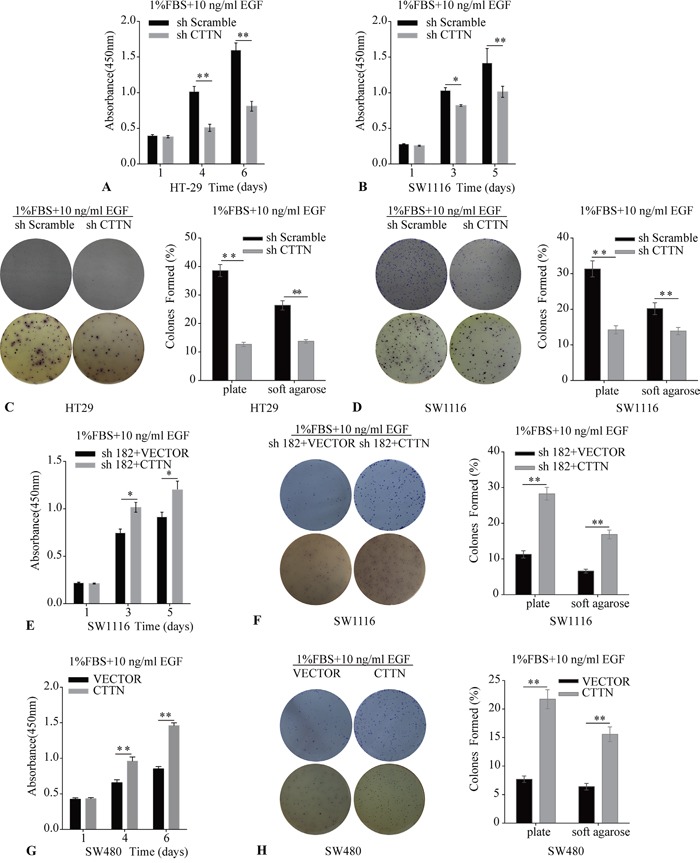
The effect of Cortactin overexpression on cell proliferation and colony formation is enhanced by EGF **A, B, C, D.** The knockdown of CTTN attenuates EGF-induced proliferation and colony formation of HT-29 and SW1116 cells. **E, F.** The rescue expression of CTTN restores the EGF-dependent proliferation and colony formation in SW1116 cells. **G, H.** CTTN overexpression enhances EGF-induced proliferation and colony formation in SW480 cells. All assays were performed in plates containing 1% FBS and 10 ng/mL EGF. Representative images from three independent experiments (*P < 0.05, **P < 0.01).

To further confirm whether the enhanced proliferative effect of CTTN was associated with EGFR protein levels, we used 10 ng/mL EGF to stimulate the CRC cells. The HT-29, SW1116 CTTN-sh, and control cells were cultured in the medium containing 1% FBS and 10 ng/mL EGF, and the relative amount of cells was measured by CCK-8 assays. The numbers of cells in the sh-Scramble groups are higher than the corresponding sh-CTTN group (Figure 6A, 6B). The up-regulation of CTTN enhances the growth of cells maintained in 1% FBS with 10 ng/mL EGF (Figure 6E, 6F). CTTN expression can promote colony formation under EGF-dependent conditions (Figure 6C, 6D, 6F, 6H). This potentiation effect of EGF indirectly suggests that CTTN may be involved in the EGFR signaling pathway which promotes CRC cell proliferation.

### CTTN overexpression attenuates EGF-induced EGFR ubiquitination and down-regulation

**Figure 7:**
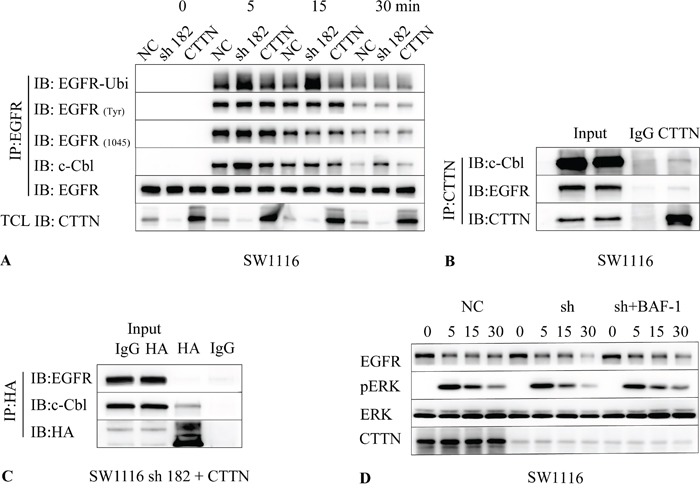
CTTN attenuates EGF-induced EGFR ubiquitination by interaction with c-Cbl **A.** SW1116 cells were pretreated with 10 μM of cycloheximide for 1 h followed by stimulation with 10 ng/mL EGF at the indicated time. The cell lysates were immunoprecipitated with EGFR and followed with anti-Ubiquitin, EGFR(Tyr), EGFR (Tyr 1045), c-Cbl and EGFR. **B.** SW1116 cells were cultured conventionally with 10% FBS, cell lysates were immunoprecipitated with CTTN, and then immunobloted with anti-EGFR, c-Cbl and CTTN. **C.** The SW1116-sh182 cells with CTTN rescue expression were lysed and immunoprecipitated with HA, and then immunobloted with anti-EGFR, c-Cbl. **D.** Serum starved SW1116 control or CTTN-sh182 cells were pretreated with 10 μM of cycloheximide and 100 μM Bafilomycin A1 (Baf-A1) for 1 h, followed by stimulation with 10 ng/mL EGF at different times. Cell lysates were analyzed with antibodies as indicated by Western Blot.

We examined the effect of CTTN depletion on EGFR ubiquitination and tyrosine phosphorylation induced by EGF. Serum starved control cells, CTTN depleted or rescue expression (CTTN-sh182 + CTTN) cells were stimulated with 10 ng/mL of EGF. Cell lysates of each group at different time points were subjected to immunoprecipitation with anti-EGFR antibody followed by immunoblotting with either anti-pTyr (totally), anti-pTyr (1045), anti-c-Cbl, or anti-ubiquitin antibodies. Upon EGF treatment, there is an overall increase in ubiquitination of EGFR in CTTN depleted cells compared with the WT group (Figure 7A). The rescue expression of CTTN also suppresses the ubiquitination of EGFR. These results indicate that CTTN expression inhibits EGFR ubiquitination induced by EGF.

Given the well-known role of c-Cbl in EGFR internalization and ubiquitination [23], we investigated whether c-Cbl is altered in CTTN-overexpressed cells stimulated with EGF. The results showed that CTTN knockdown increased binding of c-Cbl with EGFR, and rescue expression may interfere with this coupling (Figure 7A). There were no differences in the levels of totally tyrosine phosphorylation of EGFR or EGFR pTyr (1045), which is involved in c-Cbl recruitment [24]. These data imply that CTTN may interfere in the association of EGFR with c-Cbl, and inhibits the ubiquitin-mediated degradation of EGFR.

To understand the relationship between CTTN and c-Cbl, we examined whether CTTN can interact with c-Cbl. Immunoblotting of the anti-CTTN immunoprecipitates from SW1116 cell lysates revealed that c-Cbl co-immunoprecipitated with CTTN and EGFR (Figure 7B). After the cell lysates from SW1116 CTTN rescue-expression cells were subjected to immunoprecipitation with anti-HA, we analyzed the binding of c-Cbl, EGFR with CTTN. We found that HA-CTTN can interact with c-Cbl, but EGFR was minimally detected (Figure 7C). In addition, we investigated whether the suppression of ubiquitin-mediated EGFR degradation by CTNN could be blocked by treating cells with lysosomal inhibitor Bafilomycin A1 (BAF-1). SW1116 sh-182 cells treated with Baf-A1 suppressed the knockdown of CTTN-induced degradation of EGFR, as well as EGF-induced phosphorylation of ERK (Figure 7D). Attenuation of MAPK signaling may account for ubiquitin-dependent lysosomal degradation of EGFR. These results indicate that CTTN suppresses the coupling of EGFR with c-Cbl (and thereby EGFR ubiquitination), inhibits the ligand-bound EGFR for lysosomal degradation, and enhances the MAPK signaling pathway by inhibiting the association with c-Cbl.

## DISCUSSION

Colorectal tumorigenesis is a multi-step process resulting from the accumulation of genic mutations and incontrollable regulation of signaling pathways [25–28]. The *CTTN* gene is amplified in many tumors including head and neck cancer, gastric cancer, and hepatocellular cancer [11, 29–40]. CTTN was initially found as a substrate of v-Src in chicken cells [41, 42]. Subsequently, researchers established that CTTN is an F-actin binding protein that stimulates the migration and metastasis of tumor cells [43]. Our previous study also found that CTTN is overexpressed in colorectal cancer, and correlated with metastasis of colorectal cancer [44]. Here, our study further established CTTN promotes the proliferation of CRC cells *in vitro* and *in vivo*, and increases the MAPK signaling pathway by attenuation of EGF-induced EGFR down-regulation.

In addition to the well described role of CTTN in cellular motility and the formation of invasive pseudopodia [45], the proliferation effect in tumors is rarely reported. Via CCK-8 and EdU labeling assays, our laboratory established that CTTN increased CRC cell growth. We also found that CTTN promoted colony formation in plates and soft agar. The addition of EGF to culture medium enhanced the pro-proliferative function of CTTN. The phospho–antibody based proteomics array identified that the activation of ERK may promote CRC cell proliferation with CTTN overexpression. CTTN also inhibits the receptor-induced endocytosis mediated by the SH3 domain [46]. Timpson et al further demonstrated that CTTN overexpression inhibited the degradation of EGFR and c-MET in HNSCC [10, 11]. Our study found that CTTN expression in CRC cells increased EGFR protein levels, and inhibited the association of EGFR with c-Cbl. This in turn suppressed the ubiquitin-mediated degradation of EGFR, and enhanced the MAPK signaling pathway.

The association of c-Cbl with EGFR is regulated by both protein-protein interactions and tyrosine phosphorylation. The tyrosine (Y) 1045 of EGFR is necessary for the recruitment of c-Cbl [24], while the pTyr 1045 was not changed in cells with CTTN overexpression. CTTN bridges the receptor endocytic machinery with components of the actin cytoskeleton. For instance, CTTN binds the dynamin 2 that regulates the fission of endocytic vesicles, and has been found to participate in the endocytosis of receptors such as EGFR [47, 48].

Lynch, D. K. found that CD2AP (Cortactin-CD2-associated Protein), EGFR, and Cortactin co-localized in membrane ruffles induced by EGF. Cortactin links receptor endocytosis to actin polymerization, facilitating the trafficking of internalized EGFR by binding both CD2AP and the Arp2/3 complex [46]. A close relative of CIN85, CD2AP has been shown to bind both Cbl and the endophilins and thereby regulate EGFR endocytosis [49, 50]. These studies imply that CTTN may interact with c-Cbl indirectly by CD2AP. The results of our CoIP experiments demonstrate CTTN can interact with c-Cbl.

Other studies revealed that CTTN is not associated with EGFR levels, and did not have an effect on the proliferation of breast or hepatocellular cancer cells [19, 51, 52]. This observation may be due to the cellular specificity and mutations of the EGFR signaling pathway. With the emergence of EGFR mAb resistance, a combination of therapeutic targets should be considered [22]. Our study found CTTN could promote the proliferation and enhance clonogenic ability of colorectal cancer cells. Overexpression of CTTN suppresses the ubiquitin-mediated EGFR degradation that is induced with EGF, leading to sustained MAPK signaling by inhibiting the association between EGFR and c-Cbl.

## MATERIALS AND METHODS

### Cell lines and culture

Human colorectal cancer cell lines including LoVo, Caco-2, HT-29, HCT 116, SW480, SW620, SW1116 and Human kidney 293T cell were obtained from ATCC (Manassas, VA, USA). LoVo was maintained with F12K medium (SIGMA, USA); HT-29 and HCT 116 were maintained with McCoy's 5A (SIGMA, USA); SW480, SW620, and SW1116 were cultured in Leibovitz' L-15 (GIBCO, USA) with 10% FBS (GIBCO, USA); Caco-2 was cultured in MEM (GIBCO, USA) with 20% FBS (GIBCO, USA); 293T was maintained in DMEM (GIBCO, USA) with 10% FBS (GIBCO, USA). The SW480, SW620, and SW1116 were incubated at 37°C without CO_2_; the other cells were cultured in 37°C with 5% CO_2_.

### Reagents, RNAi, plasmids, package and infection

The siRNA products were designed and purchased from Gene Pharma (Shanghai, China) (Supplementary information, Table S1). We used an siRNA pool containing two different siRNA to suppress the expression of *CTTN*. The efficiency was assayed by qRT-PCR and western blot (WB). We designed one lentiviral shRNA plasmid containing two different target sites of the *CTTN*-CDS domain (CTTN-sh 273, CTTN-sh 275), and another sh 182 plasmid to target the 3′UTR of *CTTN* (Supplementary information, Table S1). The efficiency of each siRNA and shRNA was evaluated by qRT-PCR and WB.

The commercial lentiviral ORF plasmid of *CTTN* (Isoform A, the longest ORF of *CTTN*) was bought from GeneCopia (USA). The creation of Lentivirus stocks carrying CTTN-ORF or shRNA targeting CTTN followed the instructions provided by Addgene (USA). For transient transfection, cells were seeded into 6-well plates and transfected with siRNA or siRNA pool using lipofectamine 2000 (Invitrogen, USA), performed per the manufacturer's instructions. The stable cells were obtained by using the virus particles with 10~50 MOI (multiplicity of infection) to infect the cell lines, then transfected cells were selected by FACS (Fluorescence activated Cell Sorting), or by culturing with 5 μg/mL of puromycin for about one week.

### Cell proliferation assay

The growth of cells was measured with CCK-8 (Cell Counting Kit-8, Dojindo, Kumamoto, Japan). Cells were plated in 96-well culture plates (1.0~3.0×10^3^/well), and incubated for 5 to 6 days at 37°C in a humidified incubator with 5% CO_2_. Every 24h, the number of viable cells was quantified by adding 10 μL CCK-8 to each pore, and then incubated for another 2h. The absorbance of each pore at 450 nm was measured using a microplate reader. The EdU incorporation assay was used to examine cell proliferation with the Cell-Light™ EdU DNA Cell Proliferation Kit (Guangzhou Ribobio Co., Ltd, Guangzhou, China), and performed per the manufacturer's instructions. The percentage of positive cells labelled with EdU was calculated from five random fields.

### Colony formation assay

The cell suspension was prepared and plated in 6-well plates at a density of 250~1000 cells/well, with or without 10 ng/mL EGF, and incubated for 2-3 weeks. When the colonies formed after about 6 passages, the cells were then fixed with methanol containing 1% crystal violet for 15 minutes. Then the plates were washed to get rid of the remaining crystal violet, and allowed to dry.

We performed the soft agarose clonogenic assay to test the anchorage-independent growth of cells at different conditions. Agar (A9045-25G, SIGMA) at 0.6% and 1.2% in deionized water was autoclaved, and then stored in a 40°C water bath until use. Equal volumes of the two different concentrations of agar solution and 2X RMPI, F12K, or L-15 medium containing 20% FBS were mixed thoroughly, and kept at 40°C. Then 2 ml diluted 0.6% agar medium was added to 6-well plates, and allowed to cool at room temperature for about 30 minutes.

Cells were trypsinized, counted, and resuspended in the diluted 0.3% agar media, then pipetted to the bottom layer in each well immediately. The upper layer became solidified after 30 minutes at 4°C. Every 7 days for 3-6 weeks, 1 mL of culture medium containing 10 μg/mL EGF and 1% FBS was applied to the well. After colonies were visible, plates were removed, 500 μL of 5 mg/mL MTT was added, then incubated for 2 hours at 37°C. Images were captured with a digital single lens reflex camera (Nikon, JP).

### Protein extraction, western blot analysis

The cells were stimulated with 10 ng/mL EGF (Peprotech, USA) after 16 hours of serum starvation, and 1 hour of incubation with 10 μM of cycloheximide. The cells were lysed with RIPA (R0010, Solarbio), containing phosphatase inhibitor cocktail (Roche, Swiss Confederation). The protein concentration was measured by BCA assay (Thermo Scientific, USA). The cell lysates were analyzed by SDS-PAGE, transferred to PVDF membranes (Millipore Corp., USA), and then blocked with 1% BSA (Thermo Scientific, USA). The membranes were incubated with primary antibodies including Cortactin (sc-11408, SANTA CRUZ), EGFR (D38B1, CST), Phospho-p44/42 MAPK (Erk1/2, Thr202/Tyr204, CST), p44/42 MAPK (Erk1/2, CST), and GAPDH (sc-32233, SANTA CRUZ) with anti-mouse or anti-rabbit HRP secondary antibody (SANTA CRUZ, USA). The target bands were visualized by chemiluminescence (Millipore Corp., USA).

### Clinical specimens, RNA extraction, and qRT-PCR

Colorectal cancer and the adjacent non-tumor tissues (at least 5 cm away from the tumor site) were obtained from surgical specimens approved by the Ethical Committee of Ruijin Hospital, Shanghai Jiao Tong University School of Medicine. The participants were provided written informed consent to take part in this clinical study. The surgical specimens were excised immediately after cancer samples' dissection, and preserved in liquid nitrogen. The pathologic staging of the tumors was performed according to the Cancer Staging Manual from the International Union Against Cancer (7th edition, 2010).

Tumor specimens were classified into two groups based on the relative expression of CTTN/GAPDH examined by qRT-PCR. Total RNA was extracted from both cell lines and tumor tissues with Trizol reagent (Invitrogen, USA), performed per the manufacturer's instruction, and then reverse transcribed to cDNA. The cDNA was subjected to a qPCR assay for further analysis of *CTTN* mRNA levels. Primer sequences are in the supplementary information (Table S2).

### Xenograft nude mouse assay, Immunohistochemistry

Animal studies were performed as previously described [55]. For *in vivo* tumor growth assay, the stable transfected cells of SW1116 CTTN-sh 273/275 (also named CTTN-sh) were implanted subcutaneously to the left upper flank region of the male mice (1 × 10^6^ cells per mouse). The tumor size was measured weekly using calipers as follows: tumor volume (mm^3^) = (L×W^2^)/2, where L represents length, and W represents width. About six weeks later, the tumors were excised and processed for standard histological studies after the mice were euthanized. The animal experiments were approved by the Institutional Animal Care and Use Committee of the Shanghai Jiao Tong University. All animal studies were performed according to the guidelines on the care and use of animals for scientific use.

For histological analysis, the tumors of patients and nude mice were fixed in 10% formalin for at least 24 hours, and then were embedded with paraffin. The sections were stained with hematoxylin and eosin (H&E) for the morphological analysis, or with specific primary antibody of CTTN, Ki-67 (Santa Cruz, USA). The slides were stained with the 2-Solution DAB Kit (Invitrogen, USA), following the manufacturer's instructions. Based on the staining intensity of the tumor cells, staining levels were classified as negative (no or weak staining) or positive (moderate or strong staining).

We examined 61 pairs of colorectal cancer tissue by IHC and qRT-PCR. Two pathologists, who were blinded from any patient data, independently examined the cellular location of CTTN and compared the staining between the tumor and normal tissues for each case. Immunohistochemistry stain score = positive cell score + staining intensity score [53]. The percentage of positive cells was classified by five grades (percentage scores): <10% (grade 0), 10-25% (grade 1), >25-50% (grade 2), >50-75% (grade 3), and >75% (grade 4). Immunohistochemical staining intensity was graded as follows: 0 (no staining), 1 (bright yellow), 2 (orange), or 3 (brown). The total scores of ≤2, >2-5, and ≥6 were defined as negative, weak positive, and strong positive, respectively.

### EGFR PathScan array analysis

The PathScan® EGFR signaling antibody array kit (Cell Signaling Technology, USA) was used to analyze the phosphorylated molecules influenced by loss of CTTN function in the EGFR signaling pathway. This experiment was performed per procedures provided by the manufacturer. Grayscale images were further analyzed with image J.

### Statistical analysis

Data from this study is expressed as mean ± S.D. of three independent experiments, unless specified otherwise, and were evaluated with an unpaired Student's t test (two-tailed; P < 0.05 was considered significant). The CTTN mRNA expression differences between colorectal tumors and paired normal tissues were analyzed with paired Student's t-test. The χ2 test was performed to evaluate the association between the clinicopathological parameters of the CRC specimens and CTTN levels. In all statistical analyses, *P* values < 0.05 were considered significant.

## SUPPLEMENTARY FIGURES AND TABLES


