# Supplementary material for: Cortactin promotes colorectal cancer cell proliferation by activating the EGFR-MAPK pathway
Source: Oncotarget. 2016 Nov 26;8(1):1541–54. doi: 10.18632/oncotarget.13652 (PMC5352075; doi:10.18632/oncotarget.13652)
Supplement: Supplementary file 1 [file oncotarget-08-1541-s001.pdf]

Cortactin promotes colorectal cancer cell proliferation by activating the EGFR-MAPK pathway

SUPPLEMENTARY FIGURES AND TABLES

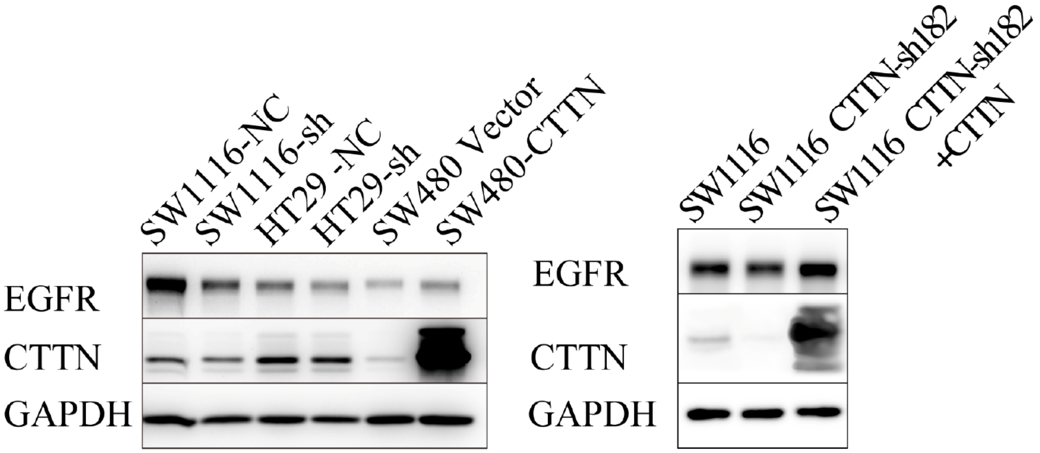

Supplementary Figure S1: CTTN expression increases EGFR protein levels in conventionally cultured CRC cells (the culture medium containing 10% FBS). The knockdown of CTTN in SW1116 or HT-29 cells decreases the protein level of EGFR, and the overexpression of CTTN in SW480 increased the protein level of EGFR. The rescue expression of CTTN in SW1116 also restored the protein level of EGFR.

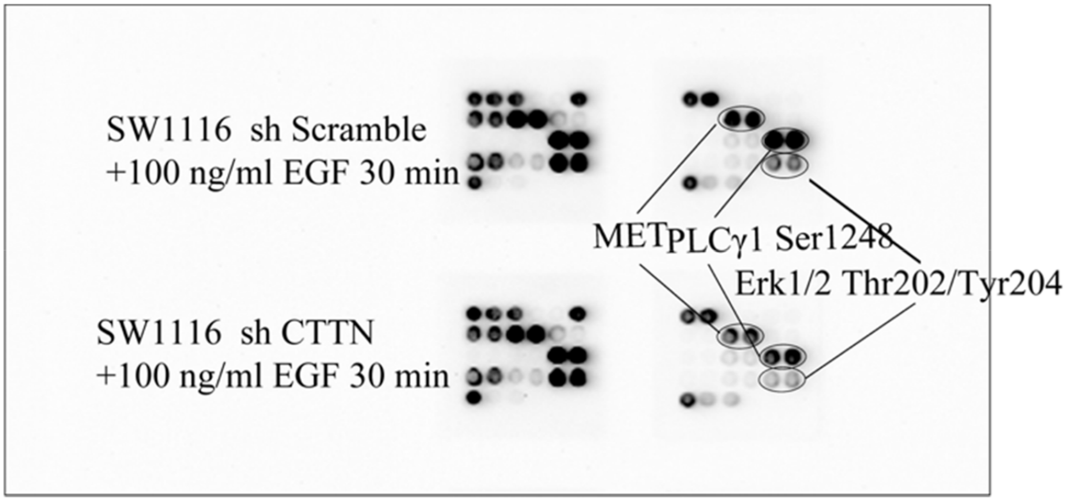

Supplementary Figure S2: The phosphorylation levels in the PathScan EGFR array of SW1116 CTTN-sh cells treated with 10 ng/ml EGF for 30 minutes.

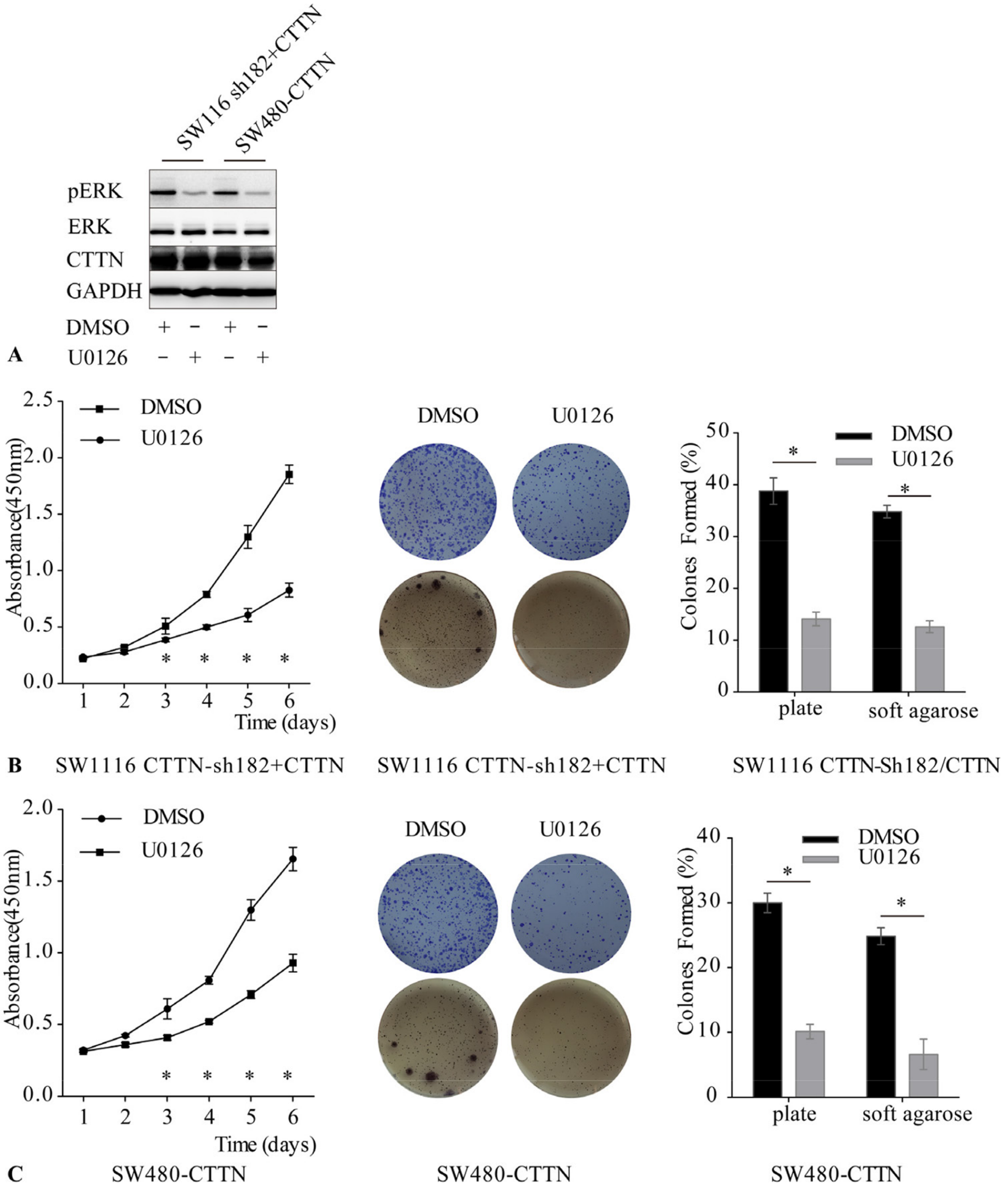

Supplementary Figure S3: CTTN promotes CRC cell proliferation via activation of the ERK1/2 pathway. A. After treatment with U0126 at the indicated times, the ERK1/2 phosphorylation in SW1116 sh-182+CTTN and SW480 CTTN cells were quantified. **B, C.** The proliferation of SW1116 sh-182+CTTN, SW480 CTTN cells were assayed in the presence of U0126 by CCK-8 and colony formation assays, $*P < 0.05$.

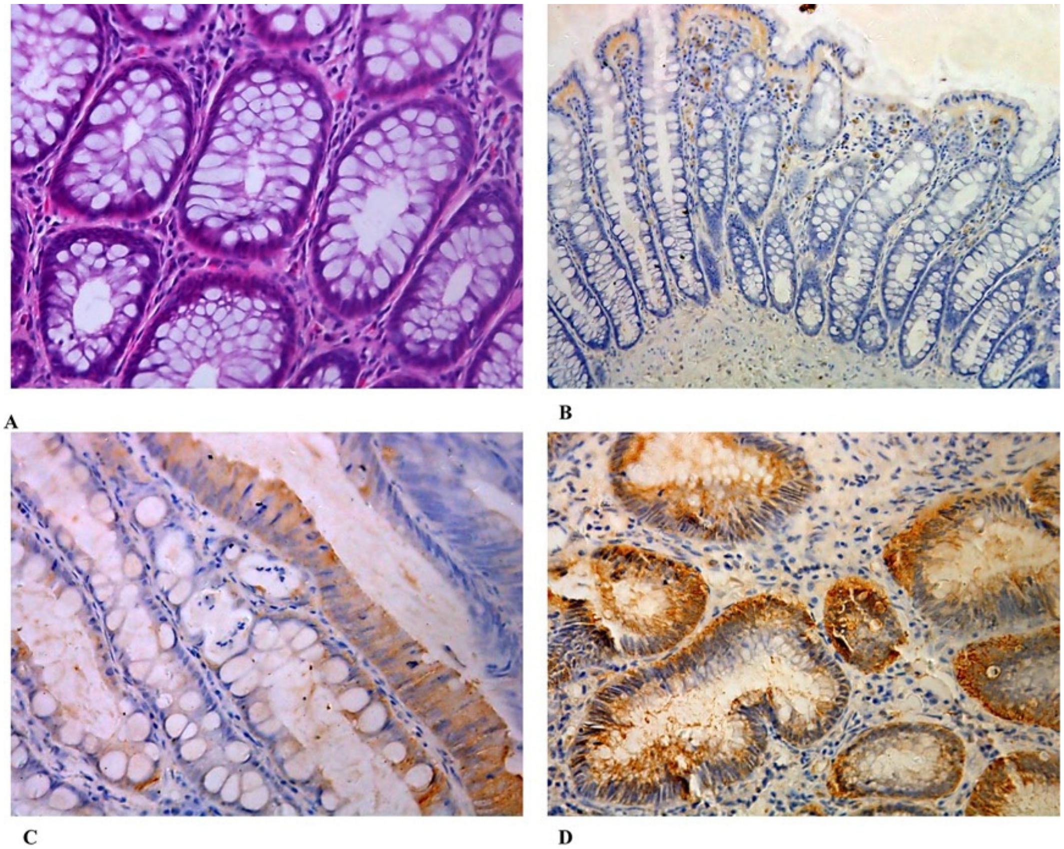

Supplementary Figure S4: Subcellular expression patterns of the CTTN. A. The HE staining of normal colorectal tissue. **B.** Immunohistochemical staining of CTTN in normal colorectal tissue. **C, D.** Representative images of weak or strong intensity staining of CTTN in CRC. (images were taken under 400×magnification)

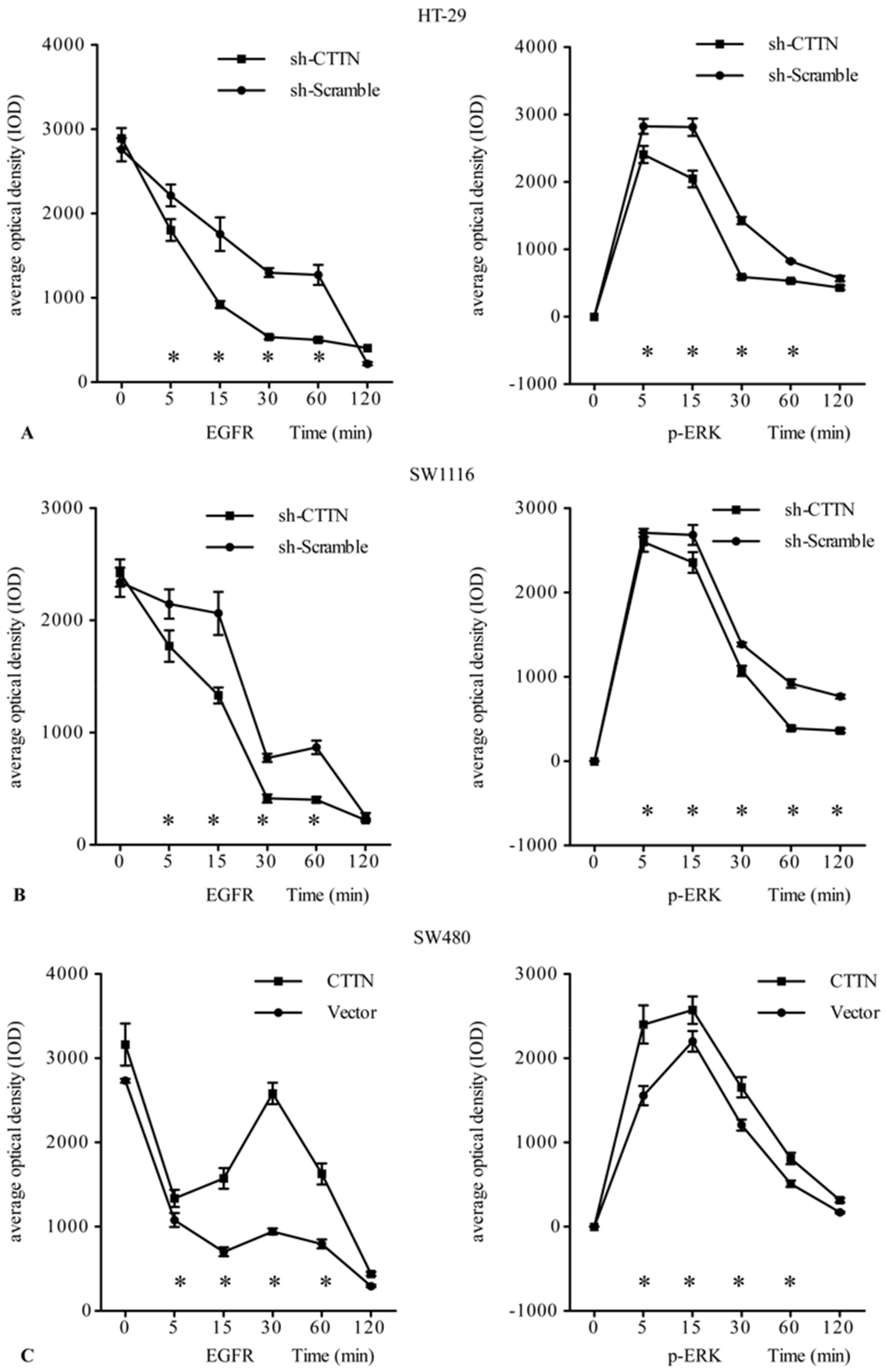

Supplementary Figure S5: Analysis of EGFR protein level and ERK activation over a prolonged time course were quantified by densitometry. A, B, C. The HT-29, SW1116 and SW480 cells were stimulated with 10 ng/mL EGFF. The quantitative analysis is corresponding with the bands of EGFR or p-ERK in Figure 5D, 5E and 5F respectively. The results are represented as average optical density (IOD) from three independent experiments. $P < 0.05$ by unpaired Student's t test, indicating differences between Cortactin-expressing cells and control cells.

Supplementary Table S1: The sequences of siRNAs and shRNA plasmids

CTTN-siRNA 377	F	GCUGAGGGAGAAUGUCUUUTT
	R	AAAGACAUUCUCCCUCAGCTT
CTTN-siRNA 499	F	CCAUGGCUAUGGAGGGAAATT
	R	UUUCCCUCCAUAGCCAUGGTT
H-CTTN-shRNA 273	F	GATCCGCGGCAAATACGGTATCGACAACTCGA GTTGTCGATACCGTATTTGCCGTTTTTG
	R	aattCAAAAACGGCAAATACGGTATCGACAAC TCGAGTTGTCGATACCGTATTTGCCGCG
H-CTTN-shRNA 275	F	GATCCGCACGAATATCAGTCGAAACTTCTC GAGAAGTTTCGACTGATATTCGTGTTTTTG
	R	aattCAAAAACACGAATATCAGTCGAAACTTCT CGAGAAGTTTCGACTGATATTCGTGCG
H-CTTN-shRNA 182	F	GATCCGCCACAGAATTTGCTAATATATCT CGAGATATATTAGCAAATTCTGTGGTTTTTG
	R	aattCAAAAACCACAGAATTTGCTAATATATC TCGAGATATATTAGCAAATTCTGTGGCG

Supplementary Table S2: Primers used for q-PCR

CTTN	F	TGGATAAGTCAGCTGTCGGC
	R	TACTTGCCGCCAAAACCACT
GAPDH	F	GGAGTCAACGGATTTGGTCGTA
	R	GGCAACAATATCCACTTTACCAGAGT
beta actin	F	ACTCTTCCAGCCTTCCTTCC
	R	TGTTGGCGTACAGGTCTTTG

Supplementary Table S3: Clinical and pathological parameters of 61 CRC specimens

Parameters	Number
Age (years)	
<60	23
≥60	38
Gender	
Male	34
Female	27
Histological type	
Tubular adenocarcinoma	56
Mucinous adenocarcinoma	5
Tumor site	
Rectum and sigmoid	41
Right colon	12
Left colon	8
TNM stage	
I	13
II	23
III	15
IV	10
pT	
T1	3
T2	12
T3	20
T4	26
pN	
N0	41
N1	12
N2	8
pM	
M0	51
M1	10

Supplementary Table S4: Correlation of CTTN expression with multiple clinical features of CRC specimens

	CTTN expression			
	High	Low	χ2	*P* value
Parameters				
Age (years)				
<60	13	10	0.011044	0.916
≥60	22	16		
Gender				
Male	22	12	1.687069	0.194
Female	13	14		
Histological type				
Tubular adenocarcinoma	32	24	0.00E+00	1
Mucinous adenocarcinoma	3	2		
Tumor site				
Rectum and sigmoid	22	19	0.751515	0.687
Right colon	8	4		
Left colon	5	3		
TNM stage				
I	4	9	10.913702	0.001
II	10	13		
III	13	2		
IV	8	2		
TNM stage				
I + II	14	22	12.277162	0.0005
III + IV	21	4		
pT				
T1	1	2	1.253189	0.263
T2	5	7		
T3	13	7		
T4	16	10		
pN				
N0	18	23	9.004228	0.003
N1	10	2		
N2	7	1		
pM				
M0	27	24	1.518921	0.212
M1	8	2		
